# Stapled EGFR peptide reduces inflammatory breast cancer and inhibits additional HER-driven models of cancer

**DOI:** 10.1186/s12967-019-1939-7

**Published:** 2019-06-18

**Authors:** Sabrina A. Maisel, Derrick Broka, Benjamin Atwell, Thomas Bunch, Robert Kupp, Shiv K. Singh, Shwetal Mehta, Joyce Schroeder

**Affiliations:** 1 Arizona Cancer Center, University of Arizona, 1515 N Campbell Ave 3945, Tucson, AZ 85724 USA; 2 Department of Molecular and Cellular Biology, University of Arizona, Tucson, AZ USA; 3 Arizona Cancer Therapeutics/Alliance Therapeutics, Tucson, AZ USA; 4 Barrow Neurological Institute, St. Joseph’s Hospital and Medical Center, Phoenix, AZ USA

**Keywords:** EGFR, HER, Juxtamembrane domain, EJ1, Peptide stapling, Inflammatory breast cancer, Glioblastoma, Lung adenocarcinoma

## Abstract

**Background:**

The human epidermal growth factor receptor (HER) family of transmembrane tyrosine kinases is overexpressed and correlates with poor prognosis and decreased survival in many cancers. The receptor family has been therapeutically targeted, yet tyrosine kinase inhibitors (TKIs) do not inhibit kinase-independent functions and antibody-based targeting does not affect internalized receptors. We have previously demonstrated that a peptide mimicking the internal juxtamembrane domain of HER1 (EGFR; EJ1) promotes the formation of non-functional HER dimers that inhibit kinase-dependent and kinase-independent functions of HER1 (ERBB1/EGFR), HER2 (ERBB2) and HER3 (ERBB3). Despite inducing rapid HER-dependent cell death in vitro, EJ1 peptides are rapidly cleared in vivo, limiting their efficacy.

**Method:**

To stabilize EJ1 activity, hydrocarbon staples (SAH) were added to the active peptide (SAH-EJ1), resulting in a 7.2-fold increase in efficacy and decreased in vivo clearance. Viability assays were performed across HER1 and HER2 expressing cell lines, therapeutic-resistant breast cancer cells, clinically relevant HER1-mutated lung cancer cells, and patient-derived glioblastoma cells, in all cases demonstrating improved efficacy over standard of care pan-HER therapeutics. Tumor burden studies were also performed in lung, glioblastoma, and inflammatory breast cancer mouse models, evaluating tumor growth and overall survival.

**Results:**

When injected into mouse models of basal-like and inflammatory breast cancers, EGFRvIII-driven glioblastoma, and lung adenocarcinoma with Erlotinib resistance, tumor growth is inhibited and overall survival is extended. Studies evaluating the toxicity of SAH-EJ1 also demonstrate a broad therapeutic window.

**Conclusions:**

Taken together, these data indicate that SAH-EJ1 may be an effective therapeutic for HER-driven cancers with the potential to eliminate triple negative inflammatory breast cancer.

**Electronic supplementary material:**

The online version of this article (10.1186/s12967-019-1939-7) contains supplementary material, which is available to authorized users.

## Background

The human epidermal growth factor receptor (HER) family consists of four transmembrane proteins (Epidermal Growth Factor Receptor/HER1/ERBB1, HER2/ERBB2, HER3/ERBB3, and HER4/ERBB4), capable of homo- and hetero-dimerizing and driving a variety of cellular activity, including migration, differentiation, proliferation, and cell survival [1–3]. HER1-3 misregulation and mislocalization are frequently associated with cancers including breast, lung, and brain [4, 5]. Given the strong inverse correlation between HER expression and progression-free patient survival, current therapies have focused on targeting either the extracellular ligand-binding domains through monoclonal antibodies or the intracellular domains through tyrosine kinase inhibitors (TKIs) [6–8]. However, these treatments often lead to therapeutic resistance driven by mutations in the kinase domain, such as EGFR T790M, which confers Erlotinib (Tarceva) and Gefitinib (Iressa) resistance in lung cancers [9, 10]. Monoclonal antibody options are also limited, as they function primarily to slow cell proliferation, allowing tumor re-growth upon removal; they also require the receptors to be at the cell surface, an event dysregulated in cancer [11–14]. Taken together, these data highlight the current limitations in targeting the extracellular or intracellular domains of HER proteins as viable long-term treatment options for cancer patients.

Despite variations in extracellular ligand binding abilities and intracellular tyrosine kinase activity, HER1-3 share homology in their juxtamembrane domain [15]. Within the juxtamembrane domain are sequences responsible for targeting HER proteins to the nucleus, binding to calcium/calmodulin, trafficking to the basolateral domain from the trans-Golgi network, and dimerization, an event required for HER activation and downstream signaling [3, 16–18]. We have previously demonstrated the efficacy of a therapeutic directed against the juxtamembrane domain region 643–663 of HER1 (EGFR Juxtamembrane Peptide 1—EJ1), capable of inhibiting HER1-3 activation, reducing calcium/calmodulin-associated proliferation, promoting cell death through necrosis and apoptosis, and reducing tumor growth and metastasis in MMTV-pyMT mice with mammary tumors [19]. However, EJ1 was degraded in less than 5 min when introduced to plasma, limiting its ability as a therapeutic. To increase the efficacy of the peptide, our current research explored the use of hydrocarbon staples to generate a stable alpha helix (SAH) of EJ1.

Native peptides are prone to losing their conformation without the support of the full-length protein to stabilize the fragment, thereby limiting their potential binding affinity and increasing the rate of proteolytic degradation [20, 21]. By introducing a stabilizing modification such as the hydrocarbon bridging of amino acid side-chains (‘staples’), the peptide can be locked into a single conformation, both chemically and structurally, leading to an increase in peptide half-life and bioavailability [22]. Stapling has been effectively utilized on multiple peptides, including a peptide designed to block the degradation of p53 and an MCL-1 inhibitor, both of which successfully induce cancer apoptosis [21, 23–25]. These peptides are not only efficacious in vitro, but a number have made it into clinical trials and patients. As of 2016, the p53-directed stapled peptide ALRN-6924 (Aileron) has progressed to Phase II clinical trials for treatment of lymphoma (NCT02264613) due to its high tolerability in patients and strong antitumor activity [26, 27]. Other cyclic peptides under clinical evaluation include POL6326 in metastatic breast cancer (Phase I completed 2018; NCT01837095) and APL-2 in age-related macular degeneration (Phase II currently recruiting; NCT03453619) [27]. Three glucagon-like peptide-1 receptor peptides have also been approved (Exenatide/Byetta [Bristol-Myers Squibb], Liraglutide/Victoza [Novo Nordisk], and Lixisenatide/Lyxumia [Sanofi]) as of 2013, with more under regulatory review and in Phase III trials [28]. We therefore investigated the hypothesis that modification of EJ1 with peptide stapling would provide the stability required for a functional therapeutic.

Here, we demonstrate the introduction of 2α,α-disubstituted residues subject to olefin metathesis followed by macrocyclic bridge formation can significantly increase EJ1 efficacy. Visualization of Cy5.5-labeled peptide demonstrated whole body delivery, with a toxic index well below the efficacious dose. When added to cancer cells with or without therapeutic resistance, we observed significant reductions in cell viability over current cancer therapies. Importantly, treatment of mouse models of breast, particularly inflammatory breast, lung, and glioblastoma resulted in decreased tumor burden and prolonged overall survival, emphasizing the therapeutic potential of a stapled EJ1 peptide.

## Materials and methods

### Peptides

**Fig. 1:**
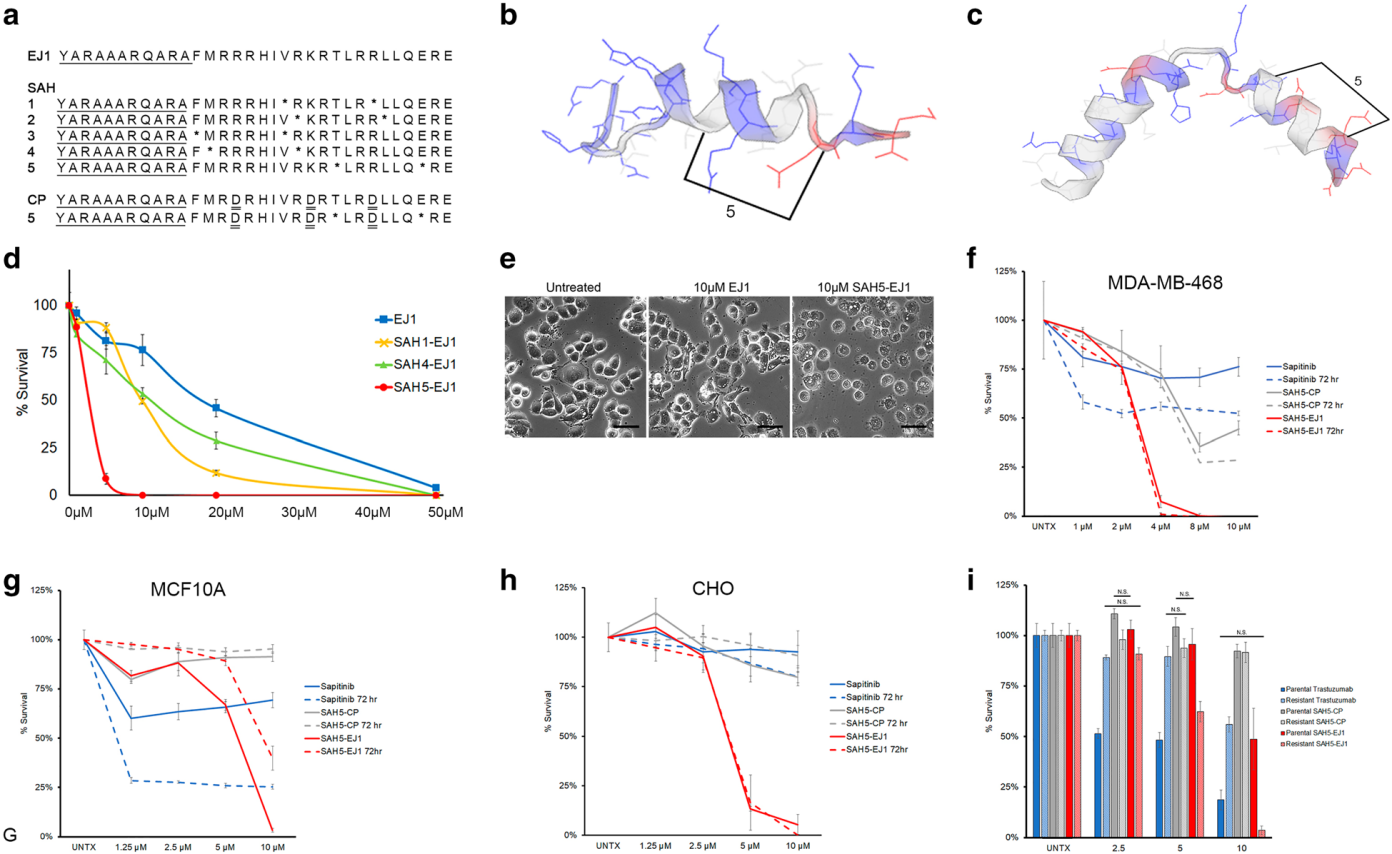
Hydrocarbon stapling leads to increased EJ1 activity in comparison to HER-directed monoclonal therapies. **a** EJ1 sequence with PTD4 domain (underlined) corresponding to EGFR juxtamembrane domain sequence 643–663. Stabilized alpha helix (SAH) variations 1–5 beneath, with asterisks (*) indicating sites of staple. Control peptide (CP) with altered residues indicated by double underline. Stapled control (5) asterisks (*) indicate sites of staple. **b** Cartoon representation of EJ1 structure, with staple variation 5 highlighted. Positively charged residues indicated in blue, negatively charged residues in red [94]. **c** Cartoon representation of CP structure, with staple variation highlighted. **d** Cell viability assay performed in MDA-MB-468 cells after 24 h comparing unstapled EJ1 versus staple variations SAH1, SAH4, and SAH5, with increasing concentrations. **e** Visualization of MDA-MB-468 cells untreated, treated with 10 µM EJ1, and 10 µM SAH5-EJ1 after 60 min. Scale bars represent 50 µm. **f**–**h** Cell viability assay performed in MDA-MB-468, MCF10A, or CHO cells over 3 days comparing treatment with Sapitinib (blue lines), SAH5-EJ1 (red lines), and SAH5-CP (grey lines). Solid lines represent 24 h; dashed lines represent 72 h. **i** Cell viability assay performed in BT474 cells over 3 days comparing treatment with Trastuzumab (µg/mL) (blue) to SAH5-EJ1 or SAH5-CP (µM) (red and grey, respectively). p < 0.001 in all cases unless indicated by NS for p > 0.05. Data shown in **d**, **f**, **g**–**i** represent mean ± percent difference of assays performed in triplicate

The peptides were synthesized as previously described [19]. The hydrocarbon staple-bearing amino acids (R-2-{[(9*H*-fluoren-9-yl)methoxy]carbonylamino}-2-methyldec-9-enoic acid and *S*-2-{[(9*H*-fluoren-9-yl)methoxy]carbonylamino}-2-methyl-hept-6-enoic acid) were added during synthesis at the positions indicated in Fig. 1. GenScript (Piscataway, NJ) performed the initial small batch synthesis of stapled peptides 1–5. The yield for numbers 2 and 3 was insufficient for testing. Large scale synthesis was carried out by PolyPeptide (Torrance, CA), and this was used for all in vivo testing. Information regarding purity and identity can be found in Table S1, Additional file 1.

### Cell lines

MDA-MB-468 triple negative breast cancer (HER1+, HER2−, HER3+, HER4−) [19, 29], BT474 breast cancer (HER1+, HER2+, HER3+, HER4+) [30, 31], BT474 clone 5 (Trastuzumab-resistant) breast cancer (HER1+, HER2+, HER3+, HER4+) [32], A549 lung cancer (HER1+, HER2+, HER3+, HER4−) [33–36], and NCI-H1975 T790M mutant lung cancer (HER1+, HER2+, HER3−, HER4−) [37, 38] cells were obtained from ATCC and cultured in RPMI-1640 medium (Corning; Corning, NY), 5% FBS (Peak; Denver, CO), and 1% Pen/Strep (Corning). BT20 triple negative breast cancer cells (HER1+, HER2−, HER3−, HER4−) [29, 30] were obtained from ATCC and cultured in Dulbecco’s modified Eagle’s medium (Corning), 10% FBS (Peak), and 1% Pen/Strep (Corning). CHO cells (HER1−, HER2+, HER3−, HER4−) [39, 40] were obtained from ATCC and cultured in F12 medium (Corning) with 10% FBS (Peak) and 1% Pen/Strep (Corning). MCF10A breast cells (HER1+, HER2+, HER3+, HER4−) [41, 42] were obtained from ATCC and cultured in DMEM/F12 medium (Thermo Fisher; Waltham, MA), 5% Donor Horse Serum (Omega Scientific; Tarzana, CA), 1% Pen/Strep (Corning), EGF (Corning), Hydrocortisone (Sigma; St Louis, MO), Cholera Toxin (Sigma), and Bovine Insulin (Fisher). SUM149 inflammatory breast cancer cells (HER1−, HER2−, HER3−, HER4−) [43] were obtained from Asterand and maintained in Ham’s medium (Gibco; Waltham, MA), 5% FBS (Peak), Insulin (Fisher), and Hydrocortisone (Sigma).

### Therapeutics

Cetuximab was purchased from Thermo Fisher (MA5-12880) and Millipore (MABF120) (Billerica, MA). Sapitinib was purchased from Selleck Chem (AZD8931) (Houston, TX). Trastuzumab was purchased from Absolute Antibody (Ab00103-10.0) (Oxford, UK).

### Fluorophore

Cyanine5.5 NHS ester (27020; Lumiprobe; Hunt Valley, MA) was conjugated to the peptide according to the manufacturers protocol. Unconjugated and precipitated dye was removed by centrifugation.

### Cell viability assay

1000–3000 cells were plated in 96-well plates (Falcon; Corning, NY) and allowed to adhere for 24–48 h prior to therapeutic introduction. Compounds were added and incubated on cells for 3 days in serum-containing media, followed by 2 h treatment with MTT (Sigma M5655) with the resulting formazan solubilized in DMSO. Absorbance was read at 540 nm by plate reader (uQuant, Bio-Tek Instruments). P-values were calculated from a one-way ANOVA.

### Live imaging

BT20 cells were treated with 150 nM MitoTracker Red CMXRos (Molecular Probes; Carlsbad, CA) for 90 min at 37 °C in serum-containing media. MitoTracker-containing media was removed, cells were treated with 5 µM Cy5.5-SAH5-EJ1 and imaged every 5 min for up to 60 min using the Leica SP5-II confocal microscope at 63× at 37 °C.

### Imaging

Immunofluorescence images were taken using a Leica DMLB microscope and Leica DFC 310 FX camera mounted on a 1× C-mount using the LAS V4.5 software. Whole Mouse imaging was performed by the Arizona Cancer Imaging Shared Resource and used LagoX (Spectral Instruments Imaging) software-AMI View (v1.7.05).

### Western blots and lysates

Tumor lysates were generated by injecting SUM149 cells into the mammary fat pads of NOD/SCID mice in Matrigel, allowing tumors to grow to 200 cubic mm and then mice were placed into arms. At the points indicated for each study, mice were dosed with the indicated quantities of SAH5-EJ1 or diluent (control arms) and monitored. Mice were sacrificed and tumor lysates were produced immediately. Cell lysates were generated as previously described [19]. Antibodies were purchased from Cell Signaling (Danvers, MA)—pCaMKII (D21E4), AKT (9272), IKKα (11930), pEGFR Y845 (2231), HER2 (2242L), Santa Cruz Antibodies (Dallas, TX)—EGFR 1005, and Sigma-Aldrich—dpERK (1/2) (M7802), β-actin (A5441). Western blotting was performed as previously described [19].

### Mouse model; inflammatory breast cancer

26 female SCID mice were injected with 0.1 mL of a cell solution containing 3.84 × 10^6^ SUM149 cells resuspended in Trevigen and sterile saline at site L4. Mice were incubated for 35 days to allow tumor growth prior to injection of SAH5-EJ1, and randomized into 3 groups, N = 8 mice/group. Injections of 5 µg/g or 0.5 µg/g in 0.1 mL volumes of SAH5-EJ1 were administered via tail vein every 3 days for 31 days until the administration route changed to intraperitoneal injections through the conclusion of the study. Control injections of equal volumes of sterile water were also performed. All animals were maintained as outlined by University of Arizona Institutional Animal Care and Use Committee (IACUC) by the Experimental Mouse Shared Resource (EMSR).

### Toxicity study

Toxicology was performed by IITRI. The study was conducted in compliance with the US Food and Drug Administration (FDA) good laboratory practice (GLP) Regulations (Code of Federal Regulations Title 21 Part 58) to evaluate the toxicokinetics, local tolerability, immunogenicity, and potential toxicity of SAH5-EJ1 following three times per week intravenous (IV) dosing for 28 days in mice. Male and female CD-1 IGS [Crl:CD1(ICR)] mice (135 per sex) were received from Wilmington, MA-based Charles River Laboratories, Inc.’s Kingston facility (Stone Ridge, NY).

### Patient-derived xenografts; breast and lung

The following PDX mouse models were generated by Jackson Labs (Sacramento, CA): Breast TM01278; Grade 3 invasive ductal carcinoma, primary malignancy; EGFR inactivating R521K mutation; ERBB3 G1288A mutation in kinase domain with unknown effects on activity; increased EGFR/ERBB2/ERBB3. Lung TM00784; Grade 3 lung adenocarcinoma, primary malignancy; EGFR activating L858R mutation; increased EGFR, decreased ERBB2/ERBB3/ERBB4. All animals were maintained as outlined by University of Arizona Institutional Animal Care and Use Committee (IACUC) by the Experimental Mouse Shared Resource (EMSR).

### Patient-derived xenografts; glioblastoma

Patient samples used were provided by the Biobank Core Facility at Barrow Neurological Institute. Samples were de-identified and conformed to the Biobank IRB protocol. Patient-derived cell lines (GB16 and GB71) were established from resected primary GBM tumor tissue. Tissue was processed using the Gentle MACS Dissociator and Tumor Tissue Dissociation kit (Miltenyi Biotec Inc.; Auburn, CA). Animal husbandry was performed according to the guidelines of St. Joseph Hospital and Medical Center and Barrow Neurological Institute under the Institutional Animal Care and Use Committee-approved protocol. Five- to six-week-old CrTac: NCr-*Foxn1*^*nu*^ nude mice (Taconic Biosciences; Hudson, NY) were used for in vivo orthotropic transplant of luciferized murine glioma model [44] (Ink4a/ARF^−/−^; hEGFRvIII). For orthotopic transplants, 2 μL of dissociated cells at a density of 100,000 cells/μL were injected in the right striatum, as described previously [44, 45]. In vivo tumor growth was measured by IVIS xenogen bioluminescence imaging (BLI) system after IP injection of 150 mg/kg Luciferin (Gold Biotechnology; St, Louis, MO) every week after 1-month post-surgery. Tumor-bearing animals were euthanized at the onset of neurological symptoms.

### Glioblastoma cell viability assay

Cells were expanded as neurospheres in tissue culture dishes coated with poly-(2-hydroxyethyl methacrylate) (Sigma-Aldrich) or grown adherent on laminin (Fisher), in DMEM and F12-Glutamax supplemented with B27 and N2 (Fisher), in the presence of 20 ng/mL EGF and 20 ng/mL FGF2 (Millipore). For dose response curves, 10,000 cells/well were plated on a laminin-coated 96-well plates. Cells were treated the following day with indicated doses of either the control peptide or SAH5-EJ1. The cell viability was assessed 48 h post-treatment with Cell Titer Glo (Promega; Madison, WI) and a Tecan plate reader.

## Results

### Hydrocarbon stapling of EJ1 increases intracellular activity

We have previously demonstrated treatment of breast tumors in vivo with a peptide directed against the juxtamembrane domain of the HER protein family (EJ1) reduced tumor growth and metastasis but was rapidly cleared in vivo [19]. To enhance peptide stability, we introduced multiple variations of hydrocarbon stapling (Fig. 1a; Table S1, Additional file 1), a chemical process which locks alpha-helices in a single native conformation [20]. Staples were oriented opposite the active face of the helix containing positively charged arginine residues, as well as away from sequences overlapping the nuclear localization sequence, the calmodulin binding domain, the dimerization domain, and the basolateral targeting sequence (Fig. 1b). Of the 5 attempted conformations, SAH2-EJ1 and SAH3-EJ1 were incapable of being synthesized. Comparison of unstapled EJ1 to SAH-EJ1 treatment on cell viability in MDA-MB-468 breast cancer cells showed that all three staple conformations enhanced the efficacy of EJ1 (IC50 = 18 µM; SAH1-EJ1 [IC50 = 10 µM]; SAH4-EJ1 [IC50 = 10 µM]; and SAH5-EJ1 [IC50 = 2.5 µM]). The most significant decrease in cell survival was observed with SAH5-EJ1 treatment (more than 7-fold; Fig. 1d). We had previously observed EJ1 induced membrane blebbing and the creation of vacuolar compartments during cell death as a part of necrosis (evaluated by the nuclear release of HMGB1), and we found a similar phenotype upon treatment with SAH5-EJ1 (Fig. 1e) [19].

We additionally created a stapled control peptide (SAH5-CP) in which the basic residues of the peptide face were replaced with acidic residues, and the peptide was similarly stapled (Fig. 1a, c). We had previously shown that single amino acid substitutions in each of the tripartite basic regions of EJ1 could alleviate function [19]. Here we demonstrate that modifying a similar peptide with hydrocarbon stapling enhanced the function of this control as well. While the control still has significantly impaired function compared to the parental peptide, it did retain some activity (Fig. 1f). In MDA-MB-468 breast cancer cells, complete cell death is achieved with 4 µM treatment of SAH5-EJ1 after 1 day, while the same concentration of the control peptide results in only 25% cell death. Doubling the concentration of this peptide results in 70% cell death (Fig. 1f).

To determine if this effect was due to the dependence of MDA-MB-468 cells on HER1 (HER1 is amplified in this cell line) [46, 47], we tested two additional cell lines; MCF10A, an immortalized breast mammoplasty cell line that expresses HER1 but not HER2 [48–50] and Chinese Hamster Ovary (CHO) cells, which express HER2 but not HER1 [51]. Relative levels of HER expression has been previously published in Hart et al. and can also be found in Figure S1, Additional file 1 [19]. Additionally, we compared SAH5-EJ1 and the stapled control peptide (SAH5-CP) to the pan-HER kinase inhibitor Sapitinib (AZD8931), selected due to its simultaneous targeting of HER1, HER2, and HER3 [52]. In MCF10A breast cells with non-amplified HER1, we found that while the cells responded to Sapitinib (demonstrating at most a 70% reduction after 72 h of treatment—dashed blue lines), complete cell death was not observed with SAH5-EJ1 until a concentration of 10 µM (Fig. 1g). Additionally, the control peptide had no significant effect on MCF10A cells at any concentration tested. Next, we compared SAH5-EJ1 and Sapitinib in HER1 negative and HER2 positive CHO cells [19]. We found that Sapitinib has limited efficacy, but SAH5-EJ1 induces significant cell death between 5 and 10 µM (Fig. 1h—red lines).

Given the efficacy of SAH5-EJ1 against HER2-expressing cell lines (13% cell viability versus 67% at 5 µM—Fig. 1g, h respectively), we next tested SAH5-EJ1 versus the monoclonal antibody therapy Trastuzumab (Herceptin) (anti-HER2 antibody), also monitoring responses in cells with therapeutic resistance to Trastuzumab. Importantly, the Trastuzumab-resistant cell lines are still capable of binding the antibody to cell surface receptors and do not present with reduced levels of HER2 expression; rather, the resistance is driven through loss of cyclin-dependent kinase inhibitor p27 expression in the nucleus [32]. Using the HER2-amplified BT474 breast cancer cell line, both Trastuzumab-sensitive (Parental) and Trastuzumab-resistant (Resistant) cells were treated with increasing doses of Trastuzumab, SAH5-EJ1, or SAH5-CP, beginning with 2.5 µg/mL (Trastuzumab) or 2.5 µM (peptides), respectively. Parental cell survival decreased to approximately 25% after 3 days of treatment with Trastuzumab (Fig. 1i, solid blue bars); as expected, Trastuzumab-resistant cells maintained almost 50% cell survival (Fig. 1i, cross-hatched blue bars). However, both parental and resistant BT474 cells demonstrated significant responses to SAH5-EJ1, particularly at 10 µM doses (resulting in approximately 50% and 5% cell survival, respectively; Fig. 1i, solid and cross-hatched red bars), highlighting the potential of SAH5-EJ1 as a therapeutic option in cancers that have become resistant to Trastuzumab. No response was observed in cells treated with SAH5-CP, regardless of Trastuzumab resistance (Fig. 1i), a trend also seen in CHO cells (Fig. 1h).

Evaluating the efficacy of SAH5-EJ1 to HER1 versus HER2 expressing cells indicate that HER1-expressing cells have increased sensitivity over HER2-expression alone. The partial response of HER1-expressing cells to the control peptide (MDA-MB-468 and MCF10A; Fig. 1f, g, respectively) versus the lack of response HER2-expressing cells (CHO and BT474) would indicate that the peptide sequence is more sensitive to HER1, given the sequence is derived from HER1 and not HER2 (despite some conserved homology within the protein family).

### SAH5-EJ1 is effectively delivered intracellularly and throughout the body

**Fig. 2:**
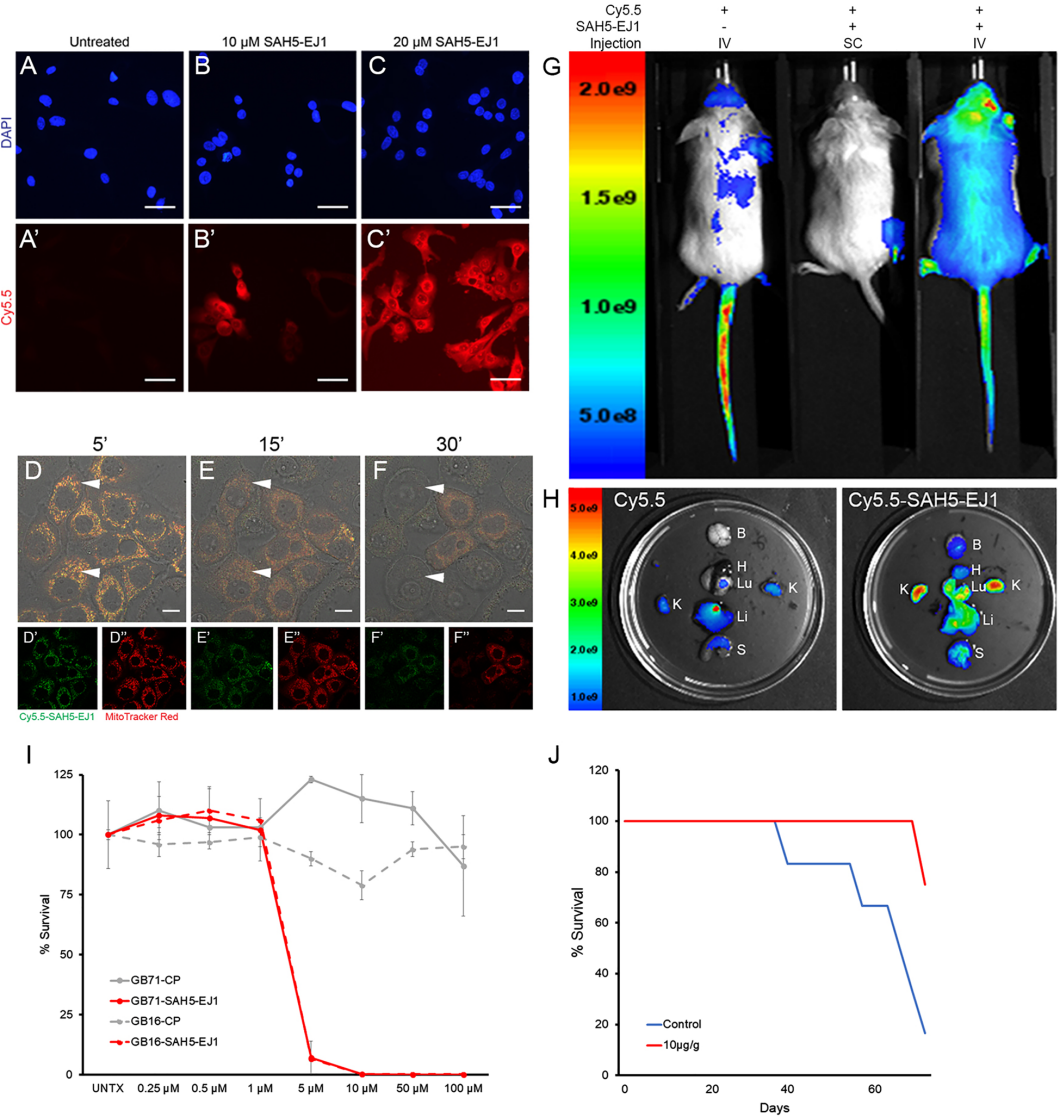
SAH5-EJ1 enters cells, colocalizes with mitochondria, and survives in vivo, resulting in glioblastoma-derived cell death and glioma mouse model survival. **A**–**C** BT20 cells treated with Cy5.5-dye. **A’** Cy5.5-only treatment, no SAH5-EJ1. **B’** 10 µM Cy5.5-SAH5-EJ1. **C’** 20 µM Cy5.5-SAH5-EJ1. Cells were incubated with Cy5.5 ± conjugation to SAH5-EJ1 (red) and mounted in DAPI (blue). Scale bars represent 50 µm. **D**–**F** Colocalization of SAH5-EJ1 with mitochondria in BT20 breast cancer cells. Cells were incubated with Cy5.5-SAH5-EJ1 (green) and MitoTracker Red (red). Single prime (‘) images represent single channel Cy5.5-SAH5-EJ1, double prime (“) images represent single channel MitoTracker Red. Arrowheads highlight changes in mitochondrial appearance. Scale bars represent 10 µm. **G** Mice were treated with Cy5.5-dye ± conjugation to SAH5-EJ1 and imaged after 30 min. Left mouse was treated with Cy5.5 dye through intravenous (IV) tail-vein injection. Middle mouse received Cy5.5-conjugated SAH5-EJ1 through subcutaneous (SC) injection in the right flank. Right mouse was treated with Cy5.5-conjugated SAH5-EJ1 through tail-vein (IV) injection. Experiment done in duplicate and representative images selected. Radiance scale provided on the left. **H** Luminescence distribution throughout organs. Cy5.5 dye only (left) versus Cy5.5-conjugated SAH5-EJ1 (right). *B* brain, *H* heart, *Lu* lung, *K* kidney, *Li* liver, *S* stomach. Radiance scale provided on left. **I** Cell viability assay performed in two glioblastoma-derived human cell lines (solid versus dashed) over 2 days comparing treatment with control peptide (CP) (grey lines) to SAH5-EJ1 (red lines). **J** Kaplan–Meier curve of glioblastoma mouse model treated with intravenous control peptide (blue) or 10 µg/g SAH5-EJ1 (red). Data shown represents mean. N = 6 for control, N = 4 for SAH5-EJ1. At the time of the GBM study, tumors formed when compound was limiting. Therefore, while enough compound was present for 4 mice, any extra mice were placed into the control arm

To determine the localization and distribution of SAH5-EJ1, the NHS ester form of the far-red Cy5.5 fluorophore was conjugated to the peptide (Cy5.5-SAH5-EJ1). Treatment of HER1-expressing BT20 breast cancer cells resulted in immediate uptake of SAH5-EJ1, with peptide distribution throughout the cell (Fig. 2A–C). We have previously discussed the ability of HER1 to be targeted to the mitochondria via the nuclear localization sequence, particularly in HER-dependent cancers [19, 53–55]. We also reported the unstapled EJ1 peptide colocalizes with the mitochondria and that this is key to reactive oxygen species (ROS) release, a primary mechanism of death by EJ1 [19]. Treating BT20 cells simultaneously with Cy5.5-SAH5-EJ1 and MitoTracker Red revealed colocalization of SAH5-EJ1 with the mitochondria within 5 min (Fig. 2D, arrowheads). Over 30 min, continued exposure of the cells to the peptide promoted a change in mitochondrial shape (Fig. 2E, arrowheads), culminating in rupturing of the cell and the mitochondria, with corresponding decreases in SAH5-EJ1 and mitochondrial luminescence (Fig. 2F, arrowheads).

To determine distribution of SAH5-EJ1 in vivo, Cy5.5-SAH5-EJ1 was injected into wildtype FVB mice using a subcutaneous (SC) or intravenous (IV) route, at 10 µg/g body weight dosages and visualized after 30 min and 24 h (Fig. 2 and Figure S2, Additional file 1). Control IV injections with the Cy5.5 fluorophore primarily displayed accumulation at the injection site in the tail (Fig. 2G, left). SC injection of labeled SAH5-EJ1 also showed a strong fluorescent signal at the injection site (Fig. 2G, middle). Alternatively, mice which received Cy5.5-SAH5-EJ1 tail vein injections presented with well-distributed peptide throughout the body, with increased concentrations in the head (Fig. 2G, right). The peptide was not detectable in plasma levels by 4 h in male mice and 2 h in female mice at the highest body weight dosage, and fluorescence was substantially reduced after 24 h (Table 1; Figure S2, Additional file 1). It is possible a maximum solubility of the drug is reached at the lowest range of dosing, resulting in no observable change in total drug plasma concentration. To identify where Cy5.5 ± SAH5-EJ1 accumulated in the mice, organs were collected post 24 h in vivo imaging and examined for Cy5.5 fluorescence directly. In mice treated with only the fluorophore, filtering organs such as the liver and kidneys displayed the highest concentrations (Fig. 2H, left). In contrast, IV-injections of Cy5.5-SAH5-EJ1 led to peptide distribution in all organs evaluated, with the highest concentrations in the kidneys and lungs, as well as observable signal in the brain, liver, and stomach (Fig. 2H, right). Taken together, these data indicate that SAH5-EJ1, upon IV injection, is distributed throughout the body and can accumulate in the brain, potentially bypassing the blood–brain-barrier.

**Table 1:** SAH5-EJ1 concentration in male and female mouse plasma

Treatment	N	SAH-EJ1 concentration (µg/mL)					
		0.5 h	1 h	2 h	4 h	8 h	24 h
Male							
Control	3	×	BQL	×	×	×	×
5 mg/kg	6	1.04	0.548	BQL	BQL	BQL	BQL
	6	1.36	0.603	BQL	BQL	BQL	BQL
	6	0.658	BQL	BQL	BQL	BQL	BQL
	Average	*1.02 ± 0.35*	*0.576 ± 0.039*	–	–	–	–
10 mg/kg	6	0.684	0.568	BQL	BQL	BQL	BQL
	6	0.514	0.589	BQL	BQL	BQL	BQL
	6	0.716	0.839	BQL	BQL	BQL	BQL
	Average	*0.638 ± 0.11*	*0.665 ± 0.150*	–	–	–	–
15 mg/kg	6	0.75	BQL	BQL	BQL	BQL	BQL
	6	1.07	0.63	0.551	BQL	BQL	BQL
	6	0.884	0.683	BQL	BQL	BQL	BQL
	Average	*0.901 ± 0.16*	*0.657 ± 0.037*	*0.551*	–	–	–
Female							
Control	3	×	BQL	×	×	×	×
5 mg/kg	6	0.561	BQL	BQL	BQL	BQL	BQL
	6	0.555	0.508	BQL	BQL	BQL	BQL
	6	0.59	0.55	BQL	BQL	BQL	BQL
	Average	*0.569 ± 0.019*	*0.529 ± 0.030*	–	–	–	–
10 mg/kg	6	0.53	0.565	BQL	BQL	BQL	BQL
	6	0.616	BQL	BQL	BQL	BQL	BQL
	6	0.539	0.518	BQL	BQL	BQL	BQL
	Average	*0.562 ± 0.047*	*0.542 ± 0.033*	–	–	–	–
15 mg/kg	6	0.508	0.588	BQL	BQL	BQL	BQL
	6	0.599	BQL	BQL	BQL	BQL	BQL
	6	BQL	0.55	BQL	BQL	BQL	BQL
	Average	*0.554 ± 0.064*	*0.569 ± 0.027*	–	–	–	–

Blood collection time point day 1 taken at 0.5, 1, 2, 4, 8, and 24 h post tail-vein injection. Average values represent peptide concentration ± standard deviation. N represents number of mice per group. × represents time points at which blood collection was not required. BQL represents below quantifiable limits (0.5 µg/mL). Three mice were bled at each time point

Italic values indicate average of SAH5-EJ1 concentrations in plasma

### SAH5-EJ1 promotes cell death in EGFRvIII glioblastoma-derived cell lines and increases survival in mouse models

Given the ability of SAH5-EJ1 to accumulate in the brains of injected mice, we wanted to evaluate its efficacy in the EGFRvIII mutant model of brain cancer. Glioblastoma multiforme is characterized as an aggressive disease driven by EGFR amplification and mutations, limited in therapies due to the challenge of crossing the blood–brain–barrier [56–58]. To evaluate the potential efficacy in this model, we treated patient-derived cell lines containing an EGFRvIII mutation in vitro. We found that SAH5-EJ1 resulted in significantly greater cell death at doses as low as 5 µM (Fig. 2I, red lines), compared to the almost 100% survival after 48 h of treatment with a control peptide (Figs. 1a; 2I, grey lines). Given this strong efficacy in vitro, we next examined the therapeutic potential of SAH5-EJ1 in an orthotopic EGFRvIII glioblastoma mouse model (Figure S3, Additional file 1) and found that treatment with 10 µg/g body weight dosages of SAH5-EJ1 significantly prolonged survival, with 75% of the mice still alive after 72 days (the only mouse death occurred after 70 days from a non-neurological disease) (Fig. 2J, red line) in comparison to only 16% of mice alive in the control peptide cohort (Fig. 2J, blue line). These data demonstrate that SAH5-EJ1 has strong therapeutic potential in EGFRvIII mutant glioblastoma and we next set out to evaluate its efficacy in lung and breast cancers.

### SAH5-EJ1 is effective in vitro and prolongs survival in PDX models of lung cancer within a therapeutic window

**Fig. 3:**
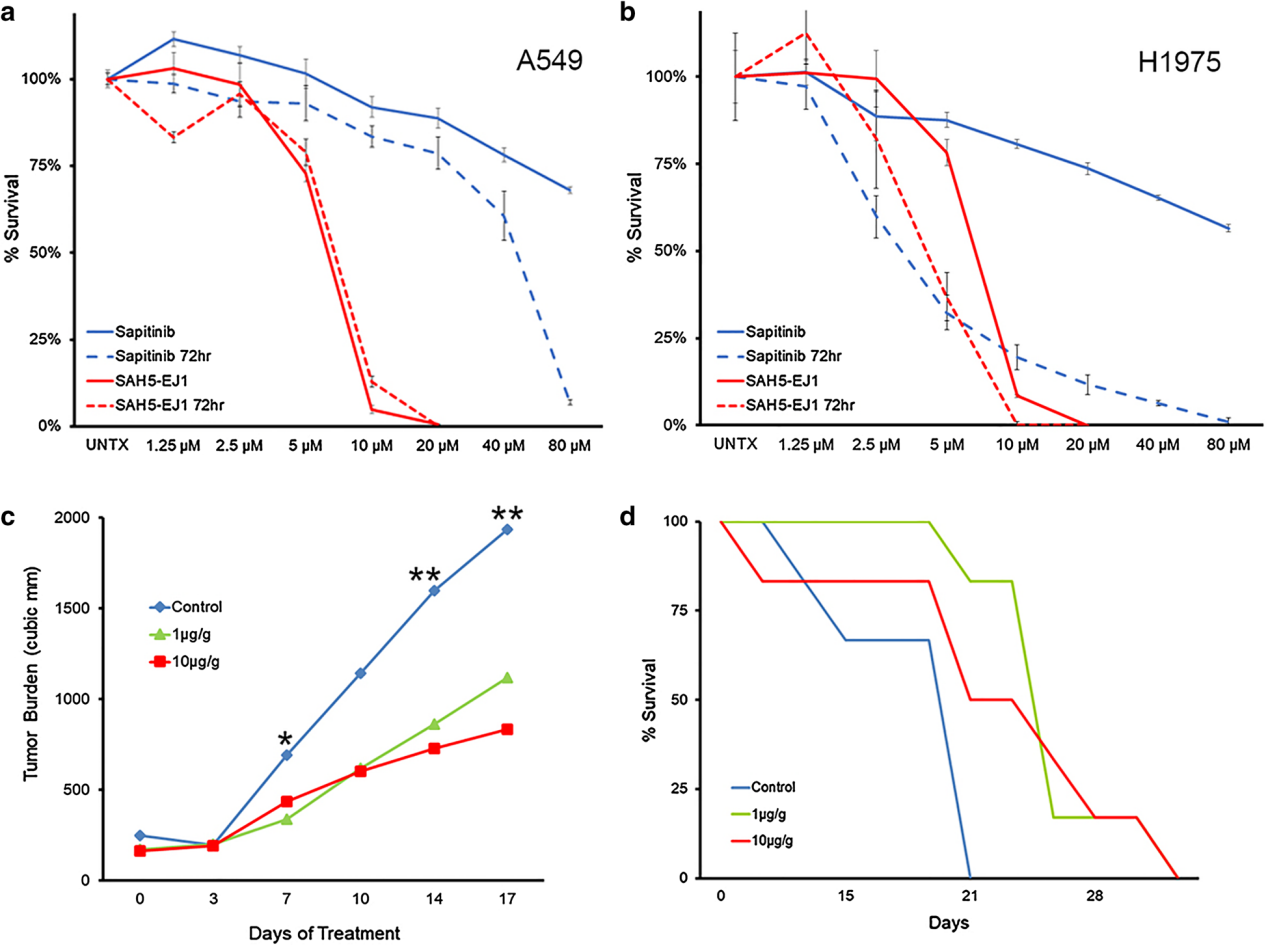
SAH5-EJ1 inhibits growth and prolongs survival in PDX models of lung cancer. **a**, **b** Cell viability assays performed in A549 or EGFR T790M mutated H1975 lung adenocarcinoma cells over 3 days comparing treatment with Sapitinib (blue lines) to SAH5-EJ1 (red lines). Solid lines represent 24 h; dashed lines represent 72 h. Data shown represent mean ± percent difference of assays performed in quadruplicate. **c** Reduction in tumor burden in Erlotinib-resistant lung cancer PDX mouse model TM00784 with various doses of intravenous SAH5-EJ1. Control represents water as a vehicle control. *p < 0.05 (Control vs 1 µg/g); **p < 0.01 (Control vs 1 µg/g and vs 10 µg/g). **d** Kaplan–Meier curve of lung PDX mouse model treated with control (blue line) or either 1 or 10 µg/g SAH5-EJ1 (green line and red line, respectively). Data shown represent mean. N = 6 for all groups

Approximately 10% of lung adenocarcinomas are driven by HER1 upregulation and kinase domain activating mutations, with HER1-targeted TKIs such as Sapitinib as standard of care [59–61]. Sapitinib is capable of targeting HER1-3, making it a potentially more potent inhibitor of HER-driven cancers and is particularly effective against HER mutants [52]. We first evaluated the efficacy of SAH5-EJ1 compared to Sapitinib in lung adenocarcinoma cell lines representing wildtype HER1 (A549) and the HER1 T790M mutation (H1975) [36, 37]. In both lines, cells were significantly more responsive to SAH5-EJ1 treatments than Sapitinib (Fig. 3a, b). This was demonstrated at both 24 and 72 h, where SAH5-EJ1 reduced cell survival to less than 5% with a 10 µM dose in the HER1 wildtype line (Fig. 3a, red lines), and 100% cell death after 72 h in the mutated HER1 line with a 10 µM dose (Fig. 3b, comparing dashed red line to dashed blue line).

We next investigated the potential efficacy of SAH5-EJ1 in the mouse model of a patient-derived lung adenocarcinoma in which the patient had been treated with the HER1 specific TKI Erlotinib and had developed resistance (TM00784). IV injection of either 1 µg/g or 10 µg/g body weight dosages resulted in more than a 50% decrease in average tumor burden (Fig. 3c). In association with the slowed tumor growth rate was a corresponding increase in survival, with mice treated with low- or high-dose SAH5-EJ1 living 1.7× longer than control-treated mice (Fig. 3d, green line and red line, respectively). There was no detected toxicity observed in the model within the therapeutic window, again emphasizing the potential therapeutic usage of SAH5-EJ1 in HER-driven cancers, even in those with resistance to other HER-directed therapies.

### SAH5-EJ1 inhibits basal models of breast cancer

**Fig. 4:**
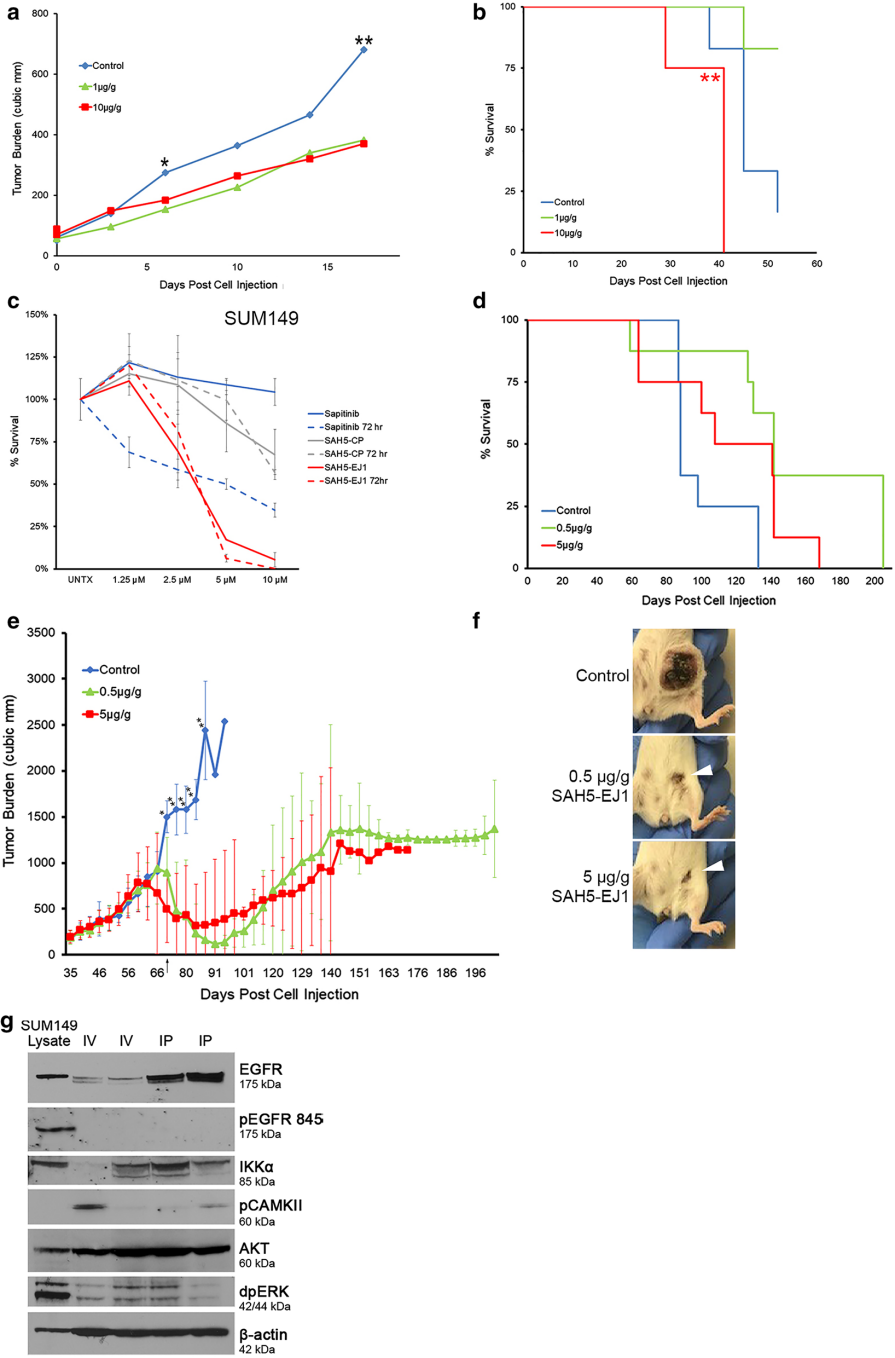
SAH5-EJ1 effectively treats models of basal and inflammatory breast cancers while increasing survival. **a** Changes in tumor burden in breast cancer PDX mouse model TM01278 with various concentrations of intravenous SAH5-EJ1. Control represents water as a vehicle control. *p < 0.05 (Control vs 1 µg/g); **p < 0.01 (Control vs 1 µg/g and vs 10 µg/g). N = 6 for each condition. **b** Kaplan–Meier curve of breast PDX mouse model treated with control (blue) or either 1 or 10 µg/g SAH5-EJ1 (green and red, respectively) (red asterisks indicate time point at which mice could no longer receive injections due to site toxicity). Data shown represent mean. N = 6 for all groups. **c** Cell viability assay performed in SUM149 cells over 3 days comparing treatment with Sapitinib (blue) to SAH5-EJ1 (red) and SAH5-CP (grey). Solid lines represent 24 h; dashed lines represent 72 h. Data shown represent mean ± percent difference of assays performed in triplicate. **d** Kaplan–Meier curve of inflammatory breast cancer SUM149-injected mouse model treated with injected control (water) (blue) or either 0.5 or 5 µg/g SAH5-EJ1 (green and red, respectively). Data shown represent mean. N = 8 for all groups. **e** Changes in tumor burden in SUM149-generated tumors with various doses of SAH5-EJ1. Control represents water (blue). Arrow indicates transition from intravenous (IV) SAH5-EJ1 injections to intraperitoneal (IP) on day 66. **p < 0.01 (Control vs 0.5 µg/g and vs 5 µg/g). Data shown represent mean ± standard deviation. N = 8 for all groups. **f** Images of mouse tumors taken on Day 116. Arrowheads indicate tumor site. Representative images selected. N = 8 for each condition. **g** Lysates were taken from tumors in mice injected with SAH5-EJ1 intravenously (IV) for 31 days of the study or mice transitioned to intraperitoneal flank (IP) injections for the remainder of the study. Control lanes represented by untreated SUM149 cells. Lysates separated by SDS-PAGE and molecular weights are indicated on the right

We next examined the efficacy of SAH5-EJ1 in a patient-derived xenograft of grade 3 invasive ductal breast carcinoma (TM01278) with increased expression of HER1 (with an R521K mutation that reduces ligand binding [62]) and increased expression of HER3 (carrying a G1288A mutation in the kinase domain [8]). SAH5-EJ1 treatment slowed tumor growth, with a significant difference in efficacy with 1 µg/g and 10 µg/g SAH5-EJ1 compared to the control treatment (Fig. 4a). We also demonstrated a 2.5-fold increase in survival when treated with 1 µg/g SAH5-EJ1 (Fig. 4b, green versus blue lines). However, all remaining mice treated with 10 µg/g SAH5-EJ1 had to be sacrificed after 41 days on the study due to toxicity at the injection site, a trend seen with increasing concentrations of the peptide (Fig. 4b, red asterisks; Table S2, Additional file 1). Taken together with basal breast cancer cell data previously introduced (Fig. 1i), these data indicate that SAH5-EJ1 can function both in vitro and in vivo for targeting breast cancers, including those with Trastuzumab resistance.

### SAH5-EJ1 effectively targets triple negative inflammatory breast cancer

Inflammatory breast cancer is an aggressive form of breast cancer, presenting with low disease-free progression and commonly associated with amplifications and overexpression of the HER proteins [63–65]. Current standard of care includes treatment with HER TKIs, often in combination with surgical resection [66–68]. However, therapeutic resistance is common, indicating a possible advantage in a HER-targeted therapeutic directed at the juxtamembrane domain.

We evaluated the efficacy of SAH5-EJ1 versus the TKI Sapitinib in the inflammatory breast cancer line SUM149 in vitro [36]. SAH5-EJ1 was significantly more effective than Sapitinib after 24 h, decreasing cell viability to less than 20% with 5 µM doses (Fig. 4c, solid red line). In contrast, Sapitinib demonstrated no reduction in cell viability when treated after 24 h, even at the highest dosage of 10 µM (Fig. 4c, solid blue line). When incubated for 3 days at 10 µM doses, Sapitinib was able to reduce viability to 35% (Fig. 4c, dashed blue line) with SAH5-EJ1 reducing viability to zero (Fig. 4c, dashed red line). These data indicate that in inflammatory breast cancer, SAH5-EJ1 is more effective at inhibiting cell survival in vitro.

We next wanted to examine the effects of SAH5-EJ1 in inflammatory breast cancer in vivo. NOD-SCID mice were injected with SUM149 cells embedded in extracellular matrix (Trevigen) in the mammary fat pad and tumors were allowed to establish (approximately 200mm^3^). IV injection occurred every 3 days (starting at day 35) for 1 month until SAH5-EJ1 administration was changed to intraperitoneal (IP) injections through the end of the study, due to reactivity at the injection site (Fig. 4e, arrow). Injection of SAH5-EJ1 (0.5 µg/g or 5 µg/g body weight dosages) resulted in a significant reduction in tumor burden, a trend maintained through the end of the study (Fig. 4e). Of note, within 55 days of treatment with SAH5-EJ1, all animals on the study showed no residual tumor burden. To evaluate the effects of treatment on overall survival, animals continued treatments for up to 203 days. By the end of the study, more than 40% of the SAH5-EJ1 treated mice remained with relatively low tumor burdens and were sacrificed upon the completion of the study, in contrast to the entire control group population which was sacrificed due to excessive tumor burden (over 2000 mm^3^) (Fig. 4d). Over time, resistance occurred, with 9/16 treated mice displaying tumor regrowth. In addition to a reduction in tumor mass, we also found the inflammatory nature of the tumor also regressed upon treatment, and the wounds healed to almost complete closure (Fig. 4f, arrowheads). Note that both 0.5 µg/g and 5.0 µg/g body weight dosages were sufficient to induce near-complete regression, with the 0.5 µg/g cohort also presenting with overall longer survival (Fig. 4e). When lysates were taken from the tumors, we observed significant reductions in total EGFR, phosphorylated EGFR, MAP Kinase (dpERK), and IKKα, while an increase total levels of AKT was also observed (Fig. 4g).

Finally, we confirmed the maximum tolerated dose of SAH5-EJ1, evaluating 5, 10, and 15 mg/kg body weight dosages (Table S3, Additional file 1). Although the injection site reactogenicity was seen following intravenous injection of SAH5-EJ1 at all three dose levels evaluated, no evidence of any systemic or target organ toxicity was seen at any dose level after a 28-day repeat toxicity study (Tables S2 and S4, Additional file 1). At 15 mg/kg, one male animal died from unknown causes and was classified as an accidental death. Therefore, 10 mg/kg was considered the no-observed-adverse-effect level (NOAEL). While the NOAEL level for SAH5-EJ1 is 10 mg/kg, tumor ablation is achieved at 0.5 mg/kg in triple negative inflammatory breast cancer, demonstrating a safe therapeutic window. Increased spleen weights observed in 10 mg/kg and 15 mg/kg-treated mice most likely resulted from extramedullary hematopoiesis, also known as “blueberry muffin baby syndrome”, an asymptomatic event in which red blood cells accumulate outside of the bone marrow [69, 70] (Table S5, Additional file 1). Increased spleen size is likely due to an increase red blood cell production associated with IV injection-affiliated cell death [71]. Clinical chemistry panels indicate that despite the high concentration of SAH5-EJ1 in the liver, liver function is not inhibited, with normal ranges of ALT/AST and total bilirubin (Table 2) [72].

**Table 2:** Liver enzyme function in male and female mouse plasma after 29 days SAH5-EJ1 treatment

	Male				Female			
	Control	5 mg/kg	10 mg/kg	15 mg/kg	Control	5 mg/kg	10 mg/kg	15 mg/kg
BUN (mg/dL)	30 ± 9.7	26 ± 2.2	31 ± 7.3	23 ± 2.9	22 ± 5.2	19 ± 2.2	19 ± 3.0	22 ± 2.5
ALP (IU/L)	54 ± 12.9	68 ± 13.8	73 ± 19.7	58 ± 10.5	95 ± 38.8	88 ± 33.2	70 ± 48.4	62 ± 18.4
ALT (IU/L)	107 ± 29.7	124 ± 71.5	96 ± 28.3	67 ± 32.3	88 ± 22.8	79 ± 35.0	61 ± 18.5	90 ± 27.2
AST (IU/L)	87 ± 28.5	92 ± 28.6	83 ± 23.6	63 ± 13.5	107 ± 16.2	80 ± 8.3^a^	83 ± 19.0	93 ± 12.5
TBIL (mg/dL)	0.1 ± 0.04	0.2 ± 0.05	0.2 ± 0.04	0.2 ± 0.05	0.2 ± 0	0.2 ± 0.05	0.1 ± 0.05	0.1 ± 0^b^
N	5	4	5	4	5	5	5	4

Blood collection for clinical chemistry analysis were collected via retro-orbital sinus puncture prior to scheduled necropsy on Day 29 and quantified using a Beckman Coulter AU480 Clinical System (Beckman Coulter; Brea, CA). Average values ± standard deviation. N represents number of mice per group. BUN blood urea nitrogen, ALP alkaline phosphatase, ALT alanine aminotransferase, AST aspartate aminotransferase, TBIL total bilirubin

^a^AST significance determined via ANOVA-DUNNETT analysis

^b^TBIL significance determined via Kruskal–Wallis-Dunn analysis

## Discussion

We have previously demonstrated the efficacy of therapeutics directed at the juxtamembrane domain of HER proteins [19]. Here we set out to determine the effects of stabilizing a peptide through α,α-hydrocarbon staples by examining changes in cancer cell viability and tumor progression. We found the stapled peptide was significantly more efficient at killing cancer cells, even when compared to current HER-directed therapeutics, both monoclonal antibodies and TKIs. SAH5-EJ1 also demonstrated increased efficacy in vivo, resulting in reduced tumor growth rates and prolonged survival. Taken together, these data indicate the improved therapeutic potential of the EJ1 peptide when stapled, particularly in comparison to standard HER-targeted cancer therapeutics.

Current therapies are limited by their dependence to single receptors or specific protein sequences, which, if mutated, can render the therapy ineffective. Studies have shown that even when treating HER2-positive breast cancer with Trastuzumab, only 35% of patients respond, due to mutations in the HER2 receptor or the activation of other tyrosine kinase pathways [8, 73]. This problem may be avoided through SAH5-EJ1 juxtamembrane domain targeting, which demonstrated significantly greater efficacy when compared to Trastuzumab treatments in vitro (Fig. 1i). Single-target therapies are also limited by HER-receptor cooperation, which promote activation of multiple downstream pathways, allowing signal transduction to remain unattenuated even with the inhibition of one of the receptors [74]. We have previously shown that using a peptide directed at the conserved domain of HER proteins promotes the formation of inactive HER dimers, overcoming this potential downfall [19].

Tyrosine kinase inhibitors are considered more effective than monoclonal antibodies as there remains more homology between HER1 and HER2 kinase domains, allowing for the potential targeting of multiple family members simultaneously [75]. However, given the proclivity of mutations to occur in the kinase domains, inhibitors have striking variations in efficacy in clinical trials, particularly in lung cancers, which present with HER2 kinase mutations in 10% of lung adenocarcinomas and HER1 T790M mutations in more than 60% of cases [76, 77]. In comparison, studies with over 2000 breast cancer subjects and 1100 lung cancer subjects found mutations in the juxtamembrane domain of HER1 in 0% and 0.07% of cases, respectively [78–81]. When compared to current TKIs, SAH5-EJ1 was significantly more effective, even in cancers with known tyrosine kinase domain mutations (Fig. 3b), emphasizing the benefits of targeting a domain in which activating mutations are clinically rare [82, 83]. Beyond the limitations currently described, the main therapeutic challenge associated with HER-driven cancers is the development of therapeutic resistance, primarily driven by increased expression of HER family members. Yonesaka et al. demonstrated Cetuximab (Erbitux; targets HER1) resistance is driven by HER2 upregulation and re-sensitizing resistant cells to Cetuximab requires inhibition of HER2/HER3 heterodimers—highlighting the importance of a pan-HER therapeutic such as SAH5-EJ1 [84]. We also observed therapeutic resistance in our models (Fig. 4e), but as we did not remove the primary tumors, we cannot speculate on the effects of SAH5-EJ1 on metastatic sites. This will be the subject of future experiments.

Glioblastoma multiforme (GBM) is associated with HER1 mutations in more than 40% of cases, particularly the EGFRvIII deletion mutation which alters the extracellular domain to inhibit ligand binding while resulting in a constitutively active conformation, an event frequently associated with therapeutic resistance [56, 85]. Given the prominent role HER1 plays in promoting GBM, HER1-directed inhibitors such as Gefitinib and Erlotinib are frequently administered but have proven unable to penetrate the blood–brain-barrier, again highlighting the limitations of current HER-directed therapies [56, 86]. As our data demonstrated efficacy against orthotopically implanted EGFRvIII PDX models, it is likely that SAH5-EJ1 can pass the blood–brain barrier, which is a subject of future studies.

Inflammatory breast cancer (IBC) is characterized by poor patient survival, high rates of metastasis, and limited targeted therapies [64, 67, 87]. Given the lack of understanding in identifying the drivers of IBC, current therapies have been wide-ranging and relatively inefficient. COX-2 (cyclooxygenase-2) has been demonstrated to be upregulated in IBC, known to moderate the production of estrogen and interact with both HER1 and HER2, driving invasion and migration in the stem-cell-like population of cancer, making it a possible therapeutic target. However, given the ubiquity of COX-2 in healthy tissues as well as cancer, high toxicity levels of COX-2 inhibitors have limited therapeutic use in clinical trials [65, 88]. Other therapies have tried to overcome upregulated Rho GTPase activity through a class of farnesyl transferase inhibitors, only to be limited by redundancies in Ras family member function and high toxicity levels [89, 90]. Nuclear transcription factor NFκB is highly upregulated in IBC, along with overexpression of HER1 and in some cases, HER2 and HER3, and we observed a decrease in the NFκB mediator IKKα when treated with SAH5-EJ1 (Fig. 4g). IBC is also characterized by increased expression of mucin 1 (MUC1), which we have previously demonstrated is responsible for altering the localization and trafficking of the HER receptors, limiting HER-driven therapeutic efficacy [12, 91, 92]. IBC can also present with upregulation of HER3, known for dimerizing with HER2 and driving resistance to monoclonal antibody therapies, indicating the need for a therapeutic that targets multiple HER family members and can generate inactive dimers, such as SAH5-EJ1 [19, 93]. Future studies will investigate the lowest efficacious doses of SAH5-EJ1, but in this present study we have illustrated the success of SAH5-EJ1 at 0.5 µg/g body weight (Fig. 4d–f), well below the level of toxicity (Table S3, Additional file 1).

## Conclusions

Through 2015, therapeutic peptides in clinical trials are dominated by those targeted at extracellular targets (more than 90%), with less than 7% designed for incs [94], indicating a clinical opportunity for a juxtamembrane-directed pan-HER therapeutic. Our data indicate that SAH5-EJ1 is a highly efficacious HER-targeted therapeutic, capable of reducing oncogenic activity in multiple cancers, both in vitro and in vivo. Additions of hydrocarbon stapling have optimized the efficacy of the peptide, including increased cell death and prolonged animal survival, allowing us to use lower concentrations within a therapeutic window ideal for the treatment of breast and lung cancers, including those with drug resistant-tumors.

## Additional file

**Additional file 1.** Additional data for novel pan-HER therapeutic. ** Figure S1.** Cell line HER protein expression. **Figure S2.** IV-SAH5-EJ1 post 24 h. **Figure S3.** Glioma-luciferase in vivo. **Table S1.** Peptide COA. **Table S2.** Injection site reactogenicity. **Table S3.** Toxicity main study. **Table S4.** Treatment observation. **Table S5.** Absolute organ weight.

## Abbreviations

ALP: alkaline phosphatase
ALT: alanine aminotransferase
ANOVA: analysis of variance
AST: aspartate aminotransferase
BQL: below quantifiable limits
BUN: blood urea nitrogen
CHO: Chinese hamster ovary cell line
COX-2: cyclooxygenase-2
CP: control peptide
DMSO: dimethyl sulfoxide
EGFR: epidermal growth factor receptor
EJ1: EGFR juxtamembrane peptide 1
EMSR: Experimental Mouse Shared Resource
ERBB: epidermal growth factor receptor
FDA: Food and Drug Administration
GBM: glioblastoma multiforme
GLP: good laboratory practice
HER: human epidermal growth factor receptor
HMGB1: high mobility group protein B1
IACUC: Institutional Animal Care and Use Committee
IBC: inflammatory breast cancer
IITRI: IIT Research Institute
IP: intraperitoneal
IV: intravenous
MTT: 3-(4,5-dimethylthiazol-2-yl)-2,5-diphenyltetrazolium bromide
MUC1: mucin 1
NOAEL: no-observed-adverse-effect level
NOD-SCID: non-obese diabetic severe combined immunodeficiency
PDX: patient-derived xenograft
ROS: reactive oxygen species
SAH: stable alpha helix
SAH5-CP: stapled control peptide
SAH5-EJ1: stapled EJ1
SC: subcutaneous
TBIL: total bilirubin
TKI: tyrosine kinase inhibitor
