# Supplementary material for: Stapled EGFR peptide reduces inflammatory breast cancer and inhibits additional HER-driven models of cancer
Source: J Transl Med. 2019 Jun 18;17:201. doi: 10.1186/s12967-019-1939-7 (PMC6582486; doi:10.1186/s12967-019-1939-7)
Supplement: Supplementary file 1 — Additional file 1. Additional data for novel pan-HER therapeutic. Figure S1. Cell line HER protein expression. Figure S2. IV-SAH5-EJ1 post 24 h. Figure S3. Glioma-luciferase in vivo. Table S1. Peptide COA. Table S2. Injection site reactogenicity. Table S3. Toxicity main study. Table S4. Treatment observation. Table S5. Absolute organ weight. [file 12967_2019_1939_MOESM1_ESM.docx]

**
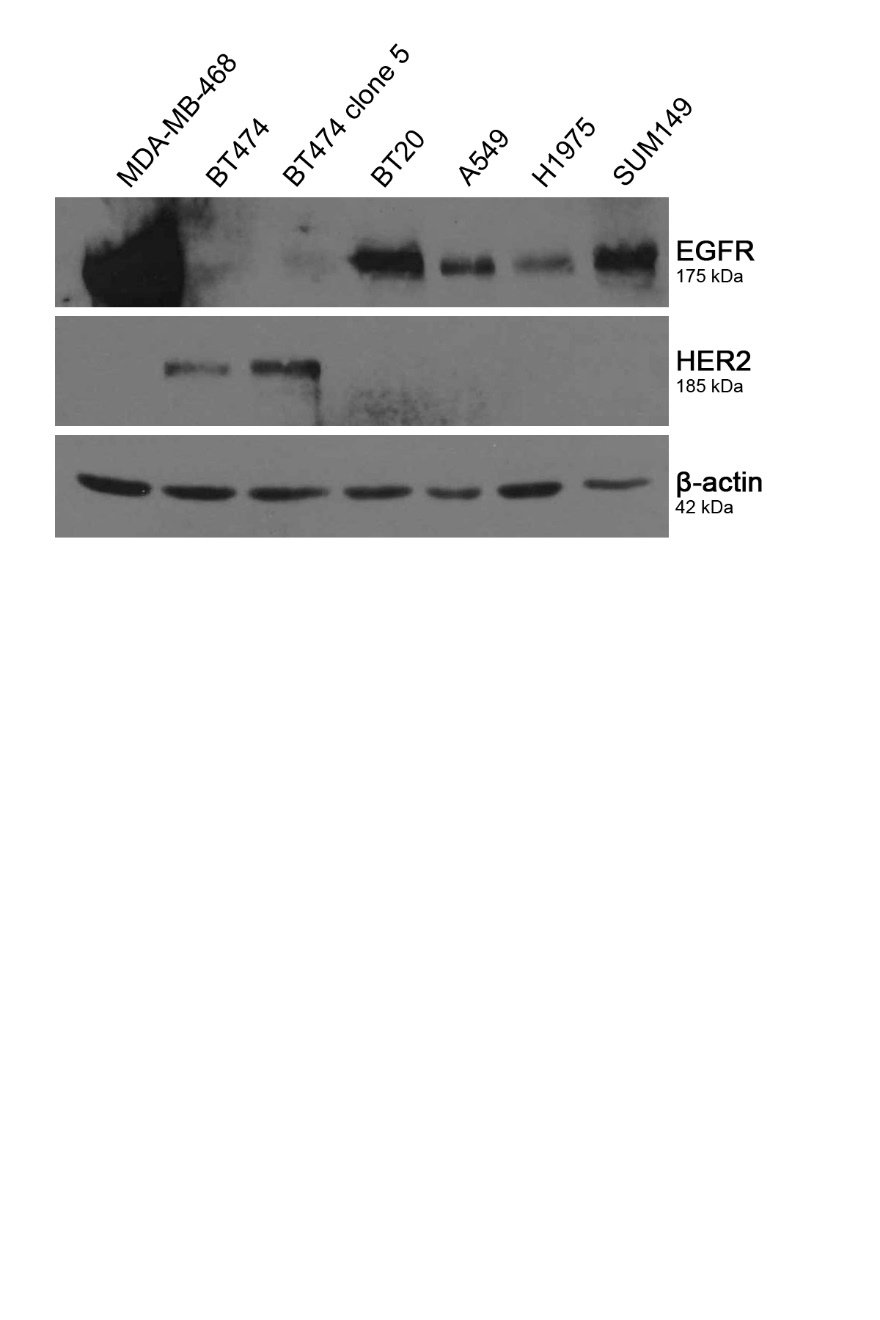
**

**Figure S1: Cell line expression of ERBB/HERs**. Lysates from MDA-MB-468, BT474, BT474 clone 5, BT20, A549, H1975, and SUM149 cells were collected and immunoblotted with EGFR, HER2, and β-actin antibodies.


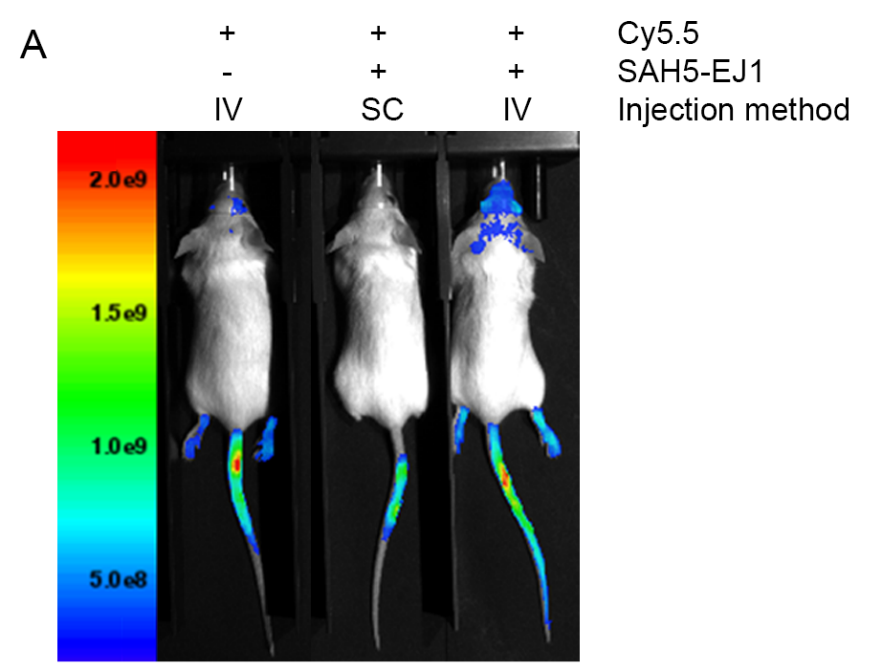


**Figure S2: IV-injected Cy5.5 ± SAH5-EJ1 remains in circulation *in vivo* after 24 hours.** **A**. Mice were treated with Cy5.5-dye +/- conjugated to SAH5-EJ1 and imaged after 24 hours. Mice were treated as described in Figure 2. Representative images selected. Radiance scale provided on the left.


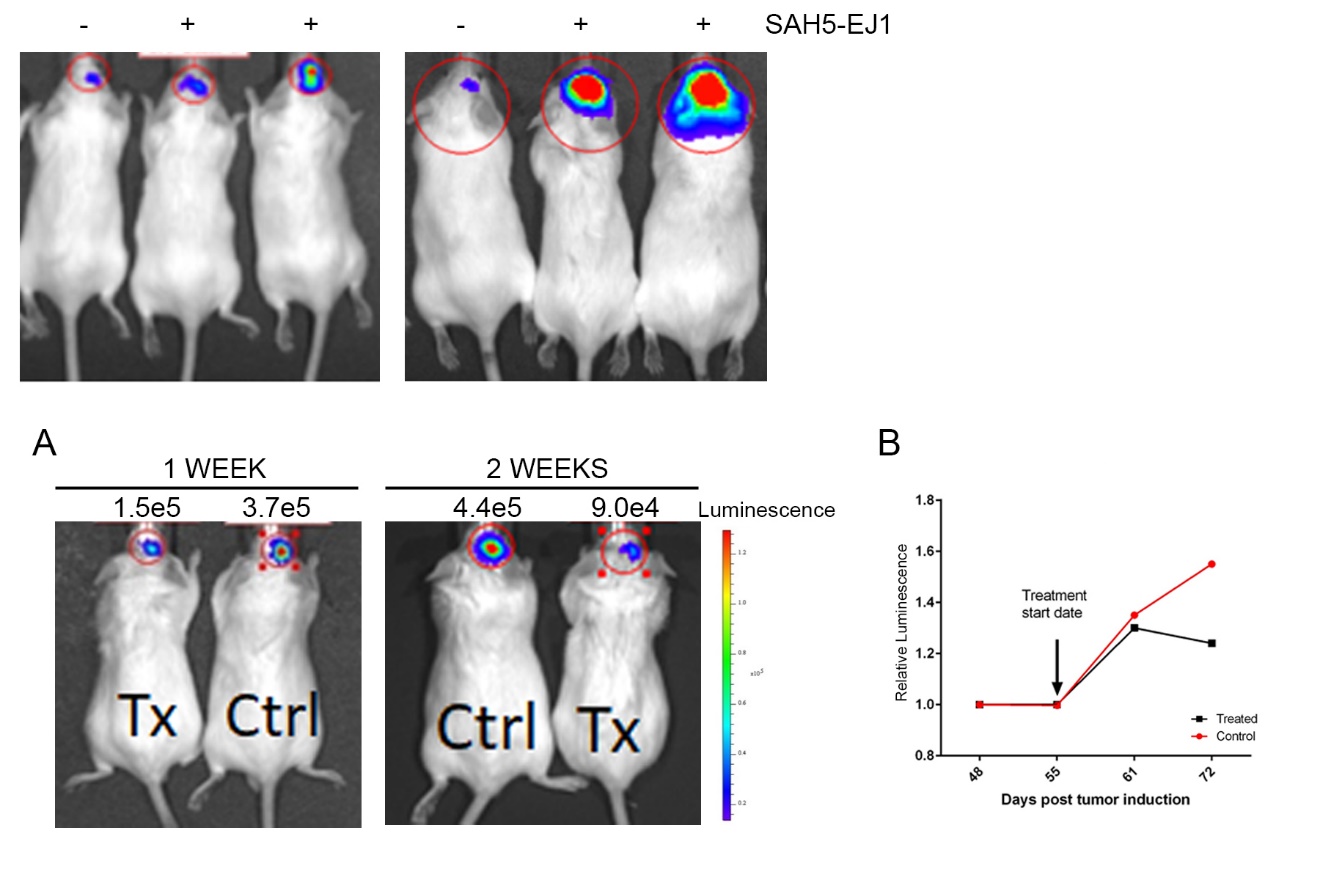


**Figure S3: Glioma tumor cell burden reduced in mice treated with SAH5-EJ1.** **A**. Mice were subjected to orthotopic transplantation of luciferized glioma cells and treated with SAH5-EJ1 one month-post injection and imaged. Ctrl indicates mice treated with SAH5-CP. Tx indicates mice treated with SAH5-EJ1. Representative images selected. Radiance scale provided on the right. **B**. Relative luminescence (compared to luminescence after 30-day initiation).

**Table S1 Certificate of analysis information corresponding to SAH5-CP and SAH5-EJ**

| **Peptide** | **Test** | **Method** | **Specifications** | **Results** |
| --- | --- | --- | --- | --- |
| SAH5-CP | Appearance | Visual Observation | Report Result | Off-white powder |
|  | Identity | MS | MW (a) = 4002.7  MW (m) = 4000.3 ± 1amu | (M+3H)^3+^/3 = 1335.3  (M+4H)^4+^/4 = 1001.9  After deconvolution: MW = 4003.3 amu |
|  | Purity Assay | RP-HPLC | ≥ 90 % | 97.3 % |
| SAH5-EJ1 | Appearance | Visual Observation | White to off-white powder | Complies |
|  | Identity | MS | MW (a) = 4098.0  MW (m) = 4095 ± 1amu | (M+4H)^4+^/4 = 1025.4  (M+5H)^5+^/5 = 820.7  After deconvolution: MW = 4098.1 amu |
|  |  | Tandem MS | Conforms to the sequence of SAH5-EJ1 | Conforms to the sequence of SAH5-EJ1 |
|  |  | AAA | Glx 2.6 – 3.5  Ala 5.1 – 6.9  Val 0.9 – 1.2  Met 0.9 – 1.2  Ile 0.9 – 1.2  Leu 2.6 – 3.5  Tyr 0.9 – 1.2  Phe 0.9 – 1.2  His 0.9 – 1.2  Lys 0.9 – 1.2  Arg 9.4 – 12.7  Correct composition ± 15% | 3.1  5.8  1.0  1.0  1.0  3.0  1.1  1.0  0.9  1.0  10.1 |
|  | Purity Assay | RP-HPLC  (Area normalized) | ≥ 90 % | 94.3 % |
|  | Related Substances | RP-HPLC  (Area normalized) | ≤ 10% | 5.7% |
|  | Acetic Acid Content | RP-HPLC | ≤ 20% | 14.4 % |
|  |  |  |  |  |
| CP (unstapled) | Identity | MS | MW (a) = 3941.4 |  |
| EJ1 (unstapled) | Identity | MS | MS (a) = 4036.5 |  |

Stapled peptide analysis performed by PolyPeptide Laboratories. Unstapled peptide analysis performed by GenScript. Abbreviations: Table MW (a) = Average Molecular Weight; MW (m) = Monoisotopic Molecular Weight; AAA = Amino Acid Analysis

**Table S2 Observed injection site reactogenicity over 28-day study**

|  | **Injection Site Reactogenicity** | | | |
| --- | --- | --- | --- | --- |
|  | **Vehicle** | **5 mg/kg** | **10 mg/kg** | **15 mg/kg** |
| **Male** |  |  |  |  |
| Animals Examined | 15 | 10 | 10 | 15 |
| Animals with Signs | 15 | 10 | 10 | 15 |
| Edema | 15 | 10 | 10 | 15 |
| 0 - Normal, no swelling | 15 | 10 | 10 | 15 |
| 1 - Minimal, slight swelling | 0 | 7 | 8 | 14 |
| 2 - Mild, defined swelling | 0 | 0 | 7 | 14 |
| 3 - Moderate, defined swelling, raised border | 0 | 0 | 1 | 7 |
| 4 - Severe, pronounced swelling, raised border | 0 | 0 | 1 | 2 |
| Erythema and Eschar Formation | 15 | 10 | 10 | 15 |
| 0 - Normal | 15 | 10 | 10 | 15 |
| 1 - Minimal | 0 | 3 | 6 | 11 |
| 2 - Mild, bright pink/pale, distinct | 1 | 2 | 8 | 15 |
| 3 - Moderate, bright red, distinct | 0 | 0 | 4 | 10 |
| 4 - Severe, dark red, pronounced | 0 | 1 | 2 | 6 |
| **Female** |  |  |  |  |
| Animals Examined | 15 | 10 | 10 | 15 |
| Animals with Signs | 15 | 10 | 10 | 15 |
| Edema | 15 | 10 | 10 | 15 |
| 0 - Normal, no swelling | 15 | 10 | 10 | 15 |
| 1 - Minimal, slight swelling | 0 | 5 | 10 | 14 |
| 2 - Mild, defined swelling | 0 | 0 | 3 | 12 |
| 3 - Moderate, defined swelling, raised border | 0 | 0 | 0 | 1 |
| Erythema and Eschar Formation | 15 | 10 | 10 | 15 |
| 0 - Normal | 15 | 10 | 10 | 15 |
| 1 - Minimal | 3 | 4 | 10 | 10 |
| 2 - Mild, bright pink/pale, distinct | 1 | 4 | 7 | 14 |
| 3 - Moderate, bright red, distinct | 0 | 0 | 5 | 5 |
| 4 - Severe, dark red, pronounced | 0 | 1 | 2 | 6 |

Response measured at 6 and 24 hours post-injection.

**Table S3 Toxicity main study animal distribution after 28 days**

|  | Study (N) | | Survival (%) | |
| --- | --- | --- | --- | --- |
| Treatment (mg/kg) | **M** | **F** | **M** | **F** |
| Vehicle Control | 10 | 10 | 100 | 100 |
| 5 | 10 | 10 | 90 | 100 |
| 10 | 10 | 10 | 100 | 100 |
| 15 | 10 | 10 | 90 | 90 |

Male (M) and Female (F) mice. Not including mice for recovery study (additional 10 mice/sex), toxicokinetics (56 mice/sex), or immunogenicity (18 mice/sex). Accidental male deaths on day 27/28 at 5mg/kg dosage and day 22/28 at 15 mg/kg dosage. Female death on day 3/28 at 15mg/kg dosage with hypoactivity noted on day 1.

**Table S4 Treatment observation frequency over 28 days**

| **Male** | **Vehicle** | **5 mg/kg** | **10 mg/kg** | **15 mg/kg** |
| --- | --- | --- | --- | --- |
| Animals Examined | 15 | 10 | 10 | 15 |
| Animals with Signs | 0 | 0 | 0 | 2 |
| Normal | 15 | 10 | 10 | 15 |
| General | 0 | 0 | 0 | 2 |
| Discolored Urine | 0 | 0 | 0 | 1 |
| Rough Coat | 0 | 0 | 0 | 2 |
|  |  |  |  |  |
| **Female** | **Vehicle** | **5 mg/kg** | **10 mg/kg** | **15 mg/kg** |
| Animals Examined | 15 | 10 | 10 | 15 |
| Animals with Signs | 0 | 0 | 0 | 2 |
| Normal | 15 | 10 | 10 | 15 |
| General | 0 | 0 | 0 | 2 |
| Hypoactive | 0 | 0 | 0 | 2 |
| Discolored Urine | 0 | 0 | 0 | 1 |
| Hunched Posture | 0 | 0 | 0 | 1 |

Frequency represents the number of animals for which a sign was observed at any point during the 28-day study

**Table S5 Absolute organ weight at terminal necropsy on day 29**

| **Male** | **Vehicle** | **5 mg/kg** | **10 mg/kg** | **15 mg/kg** |
| --- | --- | --- | --- | --- |
| Adrenal Glands | 0.017 +/- 0.0108 | 0.016 +/- 0.0097 | 0.016 +/- 0.0046 | 0.019 +/- 0.0079 |
| Brain | 0.496 +/- 0.0238 | 0.489 +/- 0.0417 | 0.503 +/- 0.0359 | 0.507 +/- 0.0396 |
| Heart | 0.247 +/- 0.0725 | 0.234 +/- 0.0448 | 0.223 +/- 0.0435 | 0.204 +/- 0.0272 |
| Kidneys | 0.622 +/- 0.0828 | 0.604 +/- 0.0683 | 0.66 +/- 0.0829 | 0.704 +/- 0.0739 * |
| Liver | 2.295 +/- 0.2542 | 2.276 +/- 0.1464 | 2.471 +/- 0.2567 | 2.393 +/- 0.2103 |
| Spleen | 0.124 +/- 0.0265 | 0.117 +/- 0.026 * | 0.179 +/- 0.0697 * | 0.192 +/- 0.0533 * |
| Testes | 0.264 +/- 0.0321 | 0.222 +/- 0.0253 | 0.237 +/- 0.0259 | 0.249 +/- 0.0456 |
| Thymus | 0.057 +/- 0.017 | 0.056 +/- 0.0092 | 0.055 +/- 0.011 | 0.045 +/- 0.0086 |
| N | 10 | 9 | 10 | 9 |
|  |  |  |  |  |
| **Female** | **Vehicle** | **5 mg/kg** | **10 mg/kg** | **15 mg/kg** |
| Adrenal Glands | 0.019 +/- 0.0052 | 0.02 +/- 0.0082 | 0.018 +/- 0.0032 | 0.021 +/- 0.008 |
| Brain | 0.498 +/- 0.016 | 0.49 +/- 0.0135 | 0.5 +/- 0.0197 | 0.5 +/- 0.0238 |
| Heart | 0.179 +/- 0.0183 | 0.189 +/- 0.027 | 0.2 +/- 0.0411 | 0.184 +/- 0.0215 |
| Kidneys | 0.393 +/- 0.0302 | 0.411 +/- 0.0379 | 0.411 +/- 0.0274 | 0.434 +/- 0.038 |
| Liver | 1.686 +/- 0.1046 | 1.726 +/- 0.0572 * | 1.771 +/- 0.1163 * | 1.92 +/- 0.2524 * |
| Spleen | 0.13 +/- 0.0215 | 0.134 +/- 0.0198 | 0.162 +/- 0.0345 | 0.205 +/- 0.0702 * |
| Thymus | 0.066 +/- 0.011 | 0.066 +/- 0.017 | 0.055 +/- 0.0132 | 0.045 +/- 0.0088 * |
| N | 10 | 10 | 10 | 9 |
|  |  |  |  |  |
| * Statistically significant difference determined by one-way ANOVA | | | | |

N represents number of mice per treatment (body weight dosage). Weight in grams +/- standard deviation. Decrease in spleen weight at 5mg/kg in males not treatment related. Increase in kidney weight at 15mg/kg in females due to slight decrease in body weight, not a reflection of treatment effects on kidneys. Increase in liver weight in females at all dosages not treatment related given a lack of dose-response trends. Thymus decrease in 15mg/kg females due to stress increases, not toxicity. ***All changes were reversible by the end of the 14-day recovery period.
